# Comparing in-phase to antiphase crew rowing: a first step from the lab to the water

**DOI:** 10.3389/fspor.2026.1604958

**Published:** 2026-04-29

**Authors:** Laura S. Cuijpers, Frank T. J. M. Zaal, Alexander Hoogerheide, Koen A. P. M. Lemmink, Harjo J. de Poel

**Affiliations:** 1 Heymans Institute for Psychological Research, University of Groningen, Groningen, Netherlands; 2 Department of Human Movement Sciences, University Medical Center Groningen, University of Groningen, Groningen, Netherlands; 3 Time Team Regatta Systems, Amersfoort, Netherlands

**Keywords:** coordination dynamics, crew rowing, group dynamics, interpersonal coordination, joint action

## Abstract

Rowing crews synchronise strokes to achieve optimal performance. Antiphase crew coordination (i.e., *alternating* strokes) may reduce velocity fluctuations of the boat, which would theoretically imply reduced hydrodynamic drag and, hence, potentially faster race times. We experimentally compared in-phase to antiphase rowing on water in terms of crew coordination and effects on boat kinematics and race time. We tested whether rowers are able to row in an antiphase pattern on the water. Next, we aimed to verify whether antiphase rowing on water indeed decreased surge velocity fluctuations and whether this would imply a higher boat speed and faster race times. Nine pairs of experienced rowers rowed four 1,000 *m* trials in in-phase and antiphase at 20 and 30 strokes per minute. Despite this being their first attempt, most crews performed the unconventional antiphase pattern stably. Antiphase rowing indeed reduced boat velocity fluctuations, especially at higher pace, but did not yield faster race times in these first attempts. The current results provide a promising first step in testing the possibilities of antiphase crew rowing on water and its effects on boat movements and velocity. Whether antiphase rowing may result in faster racing times given the potency to improve antiphase rowing through practice and optimisation of the design and rigging of the boat, will require further research.

## Introduction

Rowing is often touted as the prime example of interpersonal coordination in team sports, because of the degree of synchronisation rowers strive for to perfect their performance (1). Every stroke, the rowers collectively push off against the blades (drive) and return back to their initial position while balancing their blades above the water (recover). As a result of this discontinuous propulsion, the velocity of the boat (i.e., the shell without rowers and oars) fluctuates within the rowing cycle (2). Because the power to overcome the resistance of the water is related to the velocity of the boat to the third power [i.e., in order to row twice as fast, the rowers need to produce eight times more power (3);], it would be mechanically most efficient to maintain a constant boat velocity throughout the rowing cycle (2–7).

In theory, by rowing in an antiphase pattern a crew may be able to minimise velocity fluctuations of the boat and thereby reduce the associated power losses with 5%–6% (2–4). The boat is propelled more continuously through the water, as the rowers alternate their propulsive phases with each other. For a double scull (a two-person crew in which each rower handles two oars), this would imply that if the bow rower (rower 1) would be able to perfectly align their drive with the recover of the stroke rower (rower 2) and vice versa, propulsive force would be applied continuously on the water. Furthermore, they would also move their bodies and oars towards and away from each other, reducing the crew's combined centre of mass displacement relative to that of the boat. As such, it has been suggested that rowing in antiphase (i.e., alternating strokes) may reduce hydrodynamic drag and potentially yield faster racing times (4–7).

While from a mechanical perspective rowing in antiphase may be more efficient than in-phase rowing, it has been argued from a coordination dynamics perspective that rowing in antiphase may be more challenging than in in-phase coordination (1, 5, 8). Studies on cyclic interpersonal coordination tasks demonstrated that the stability of antiphase coordination is lower than that of in-phase coordination (9–11) and that if movement frequency increases, the stability of coordination decreases, even more so for antiphase coordination (10, 11). At a critical frequency, antiphase coordination becomes unstable and a transition to the remaining stable in-phase coordination pattern may occur (12, 13), which in the context of rowing would be detrimental for race performance. As in crew rowing stroke rates can reach up to 46 strokes per minute (*spm*) in on-water racing, antiphase coordination needs to remain sufficiently stable at high movement rates in order to be successful in competition.

Recent lab studies (5, 6, 8), in which pairs of rowers rowed in in- and antiphase patterns on coupled ergometers on slides (allowing them to move with respect to the ground), showed that rowers are able to row stably in antiphase coordination on ergometers, also at the higher (racing) stroke rates (5, 8). In addition, the movements of the coupled ergometer system, reflecting boat velocity fluctuations in de laboratory set up, drastically diminished when the rowers rowed in antiphase compared to in-phase coordination (5, 6). Moreover, the rowing pairs were able to produce similar power in antiphase as in in-phase coordination (6). The latter is important, because even if rowing in antiphase would reduce power losses, it would not be effective if it also limited power production (3, 14). Together, the lab results suggest that rowing in antiphase coordination might indeed have the potential to improve race times compared to rowing in in-phase coordination.

After promising results from these lab studies, we now set out to test the potential benefits of antiphase rowing in an experiment on the water. To date, no controlled on-water experimental study has directly tested antiphase crew coordination and the effects thereof on boat movements and velocity. An important first step is to test whether rowers are also able to row in antiphase in (changing conditions of) water and wind, when they have to propel a boat forward through the water, while many other things (e.g., pressure on the blades) also alter compared to the normal in-phase rowing. If so, does rowing in antiphase indeed reduce velocity fluctuations of a boat that is moving through the water and does this result in higher average velocity and hence faster racing times?

## Method

### Participants

Eighteen rowers with at least one year of competition experience on national level, paired in nine crews, participated in the experiment (8 women, 10 men; age 23 ± 5 years; body height 1.83 ± 0.09 *m*; body mass 75 ± 11 *kg*; rowing experience 6 ± 4 years; all rowers had experience with sculling, i.e., rowing with two oars). Participants provided written informed consent. The local Ethics Committee approved the study that was conducted according to the principles expressed in the Declaration of Helsinki. For more detailed information on the different pairs, see Supplementary Table 1 in Supplementary Materials.

### Experimental set up

Trials were performed in a quad (i.e., four-persons rowing boat, in which each rower is handling two oars; see Supplementary Table 2 for rigging and specifications of boat and oars), leaving the two middle seats empty to allow for sufficient space for the oars not to collide. Using a four-person instead for a two-person boat was necessary for feasibility reasons, but has implications for the results obtained, e.g., rowers need to propel a relatively heavier and longer shell (see Discussion). The horizontal angles of the oars, reflecting the stroke movements of both rowers, were measured using potentiometers (Bourns, 6639 Precision Potentiometer, 200 *Hz*). Movements of the boat in terms of linear accelerations and angular velocities were sampled with a three-axial accelerometer-gyroscope sensor (MPU-6050, InvenSense Inc., 200 *Hz*). Longitude and latitude of the boat was registered using a GPS sensor (PmodGPS, Digilent Inc., sampling rate: 1 *Hz*) but not used in further analysis. These sensors were placed in a waterproof housing secured to the boat behind the bow rower (i.e., in direction to the bow of the boat). The outputs of these different sensors were sampled on a microcontroller (MyRio-1900, National Instruments) and written onto a SD-card within the waterproof housing. The 1,000 *m* trials were timed using a stopwatch (Fasttime 14). As an estimate to control for potential differences in effort between conditions, heart rate of both rowers was measured using a heart rate monitor (Polar M400, 1 *Hz*).

### Protocol

To warm up, each pair started rowing towards the start of the race course, practicing the antiphase- and in-phase rowing pattern for equal bouts of time (about 15–20 *min* warming up at 18–22 *spm*). The pairs performed four 1,000 *m* trials, two in in-phase and two in antiphase with a running start, so that the boat was up to speed when crossing the starting line. Pairs rowed the four conditions in counterbalanced order, starting in either in- or antiphase and at 20 or 30 *spm*, but always alternating both pattern and stroke rates in subsequent trials to minimise effects of fatigue. Each pattern was performed both at 20 *spm* (endurance training) and at 30 *spm* (racing rates). Note that the current set up with two rowers rowing a four-person boat limits the maximum stroke rate that can be achieved. During pilot testing we asked two heavyweight males to do a maximum sprint in the current set up. They reached 36 *spm* as maximum. Because pairs would have to row two times 2000m at racing speed and we decided to set the stroke rate for the racing condition at 30 spm.

These trials were performed on the same part and in the same direction of the course to minimise influences of current and wind. Changes in weather between conditions within a session were checked and remained stable within pairs. The rowers rowed back 1,000 *m* to the start of the course after each trial in the same pattern as the following trial would be. At the start and at the end of the trial there was a break of 30 *s* in which rowers had to keep their oars perpendicular to the boat, with the blades resting on the water. This was done to (re-)determine initial values of the accelerometer- and gyroscope sensor and the oar angles. Rowers were instructed to maintain a comparable intensity in both the in- and antiphase patterns and received feedback about their heart rate on a heart rate monitor. The stroke rower (i.e., the rower crossing the finish last) received real-time feedback about stroke rate on an on-board computer with a small display (Speed Coach GPS-II, Nielsen Kellerman, US).

### Data analysis

Kinematic time series were analysed using customised procedures in Matlab (MathWorks, USA). The time series were corrected for initial position of the sensors using the average sensor values as obtained in the first 30 *s* rest bin (see above). The time series were interpolated using a piecewise spline and were low-pass filtered using a bi-directional second order Butterworth filter with a cut-off frequency of 4 *Hz* for the oar angle time series, 20 *Hz* for the accelerometer time series and 15 *Hz* for the gyroscope time series (14, 15). After this, the sensor values in *mV* were converted into oar angles (°), linear accelerations (*m/s^2^*) and angular velocities (*°/s*). For further analysis, for all pairs, steady state bins of 60 rowing cycles were determined for each 1,000 *m* trial (see below). The spatio-temporal relation between the rowers’ oar angles for both starboard and port (reflecting interpersonal coordination) around the catch was expressed by the discrete relative phase (5, 8, 14, 16). The discrete measure of relative phase based on point-estimates of oar angle extrema near the catch and finish of the stroke was calculated for each full cycle as (Equation 1):

$$\phi_{PE}(t)=\frac{t_{2,j}-t_{1,j}}{t_{2,j+1}-t_{2,j}}360^{\circ }$$ (1)

in which *t*_1*,j*_ and *t*_2*,j*_ indicate the time of the *j^th^* peak of the oar angle of starboard and port or rower 1 and 2. The instances of the catch and finish were determined as the minimum (catch) and maximum (finish) excursions of the oar angle signal as derived using a custom made peak-picking algorithm.

### Dependent measures

For all trials, deviations from steady state (coordinative breakdowns), defined as a deviation of relative phase value ≥ 180*°* of the instructed pattern (0*°* for in- and 180*°* for the antiphase pattern) for at least one complete movement cycle, were counted [see (8)]. Next, for the steady state trials (in which no coordinative breakdown occurred), the time series were analysed over steady state bins (60 *cycles*) for each condition. As measures of coordinative performance, for each trial we determined mean absolute error (*AE*) with respect to the instructed pattern and standard deviations (*SD*) of discrete relative phase (*AEϕ_catch_* and *SDϕ_catch_*). As the drive and recover differ in duration (14, 17), especially at lower stroke rates, which is likely to influence movements of the boat, the drive-recover ratio (*ratio*) is reported. Movements of the boat are reported in terms of fluctuations (i.e., standard deviations) of surge and heave (based on the accelerometer time series) and pitch and roll (based on the gyroscope time series), as represented by *SDsurge, SDheave, SDpitch* and *SDroll*^14^. Finally, race times and average heartrate (to control for differences in effort between in- and antiphase) for each rower are reported for each condition.

### Statistical analysis

Each of the above-mentioned steady state dependent measures was subjected to a 2 Pattern (in- and antiphase coordination) × 2 Tempo (20 and 30 *spm*) repeated measures ANOVA. An *α* of 0.05 was adopted for all tests of significance. If necessary, interaction effects were further scrutinised via Bonferroni-corrected (*α* of 0.0125) *post-hoc* paired-samples *t*-tests, with effect size depicted by Cohen's *d*.

## Results

**Figure 1:**
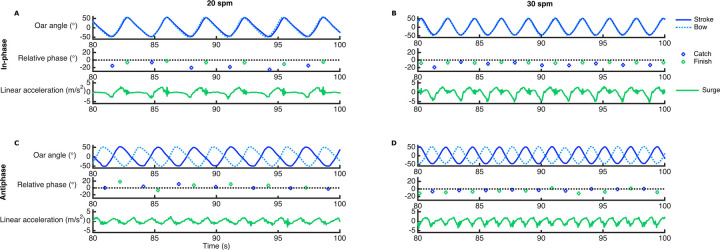
Example of kinematic time series for the different experimental conditions: in-phase [upper panels, see **(A)** and **(B)**] and antiphase [lower panels, see **(C)** and **(D)**] at 20 [left panels, see **(A)** and **(C)**] and 30 spm [right panels, see **(B)** and **(D)**]. Each panel depicts the oar angles of both rowers (subplots 1), the discrete relative phase with respect to the intended pattern around catch and finish (subplots 2) and movements of the boat in terms of surge velocity fluctuations (subplots 3).

The rowers in the present study had never rowed in antiphase before. Still, already at these first attempts they managed quite well to row in this pattern. Seven out of the nine pairs were able to perform all trials, with two pairs showing only two incidental breakdowns from the antiphase rowing pattern. During these two incidental breakdowns, the pairs were able to immediately restore crew coordination within the next rowing cycle, and continued rowing in antiphase for the rest of their trial. In two different pairs, antiphase coordination broke down repetitively within their 30 *spm* trial, while at 20 *spm* no breakdowns occurred. For reasons of fair comparison of steady-state behaviour over conditions, the subsequent results on boat movements, race time and crew coordination are based on the five pairs that did not show any coordinative breakdowns (for mean values and standard errors, see Supplementary Table 3). Note that this results in a reduction of statistical power and thus generalisability of the findings. An example of the obtained kinematic time series for the different experimental conditions is shown in Figure 1.

### Crew coordination

**Figure 2:**
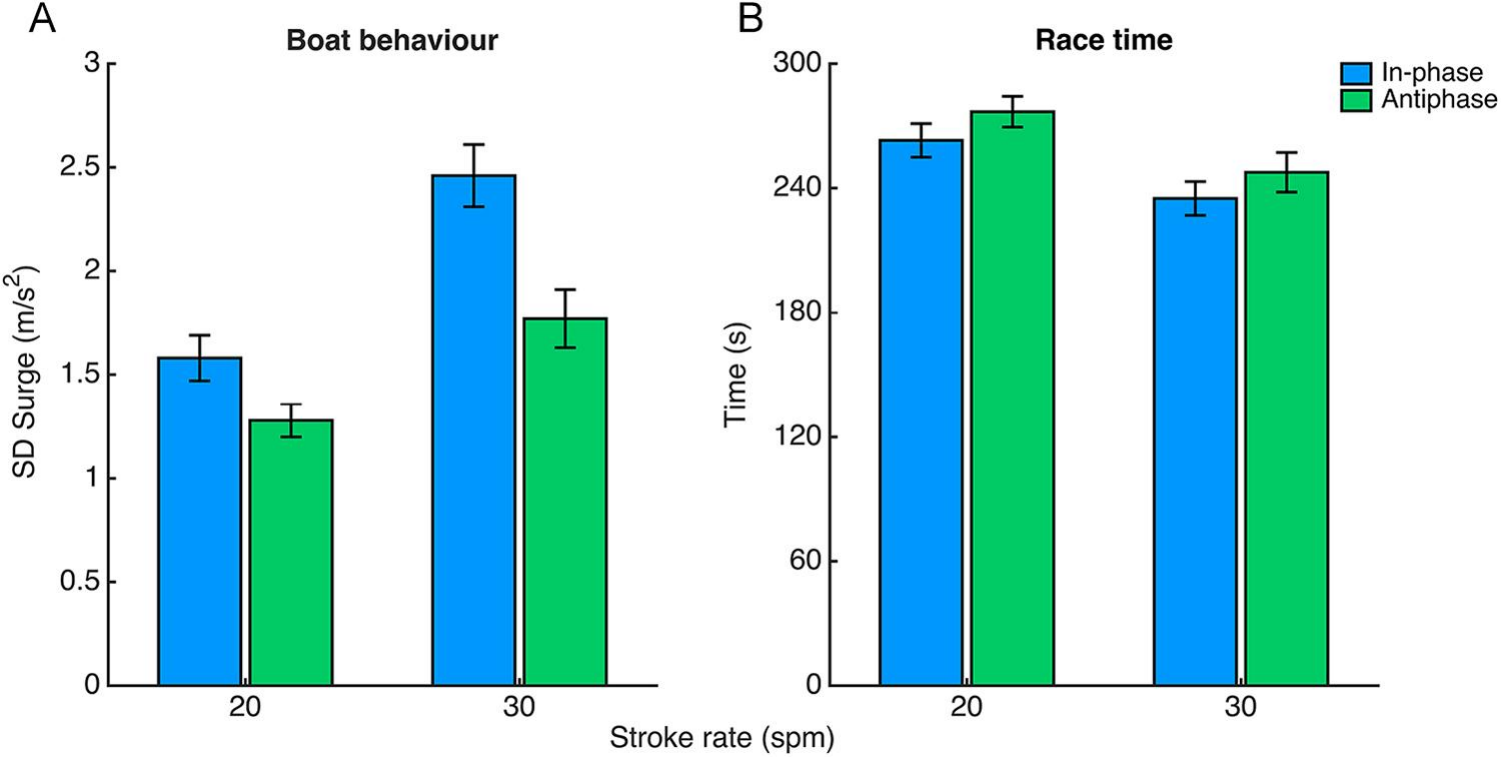
Average values of variability **(A)** and accuracy **(B)** of crew coordination and around the catch for in- and antiphase at 20 and 30 spm, as determined over 5 out of 9 pairs (see ‘coordination breakdowns). Error bars represent standard errors.

The results on the variability of relative phase at the catch (*SDϕ_catch_*) for the different conditions are displayed in Figure 2A, which clearly shows that crew coordination was less variable for the in-phase pattern and the higher stroke rate. Indeed, *SDϕ_catch_* and *SDϕ_finish_* were significantly affected by both Pattern and Tempo, indicating more variable coordination for the antiphase pattern and the lower stroke rate. For *SDϕ_catch_* the effect of Pattern was *F*(1,4) = 21.06, *p* = .01, *η_p_^2^* = .84 and the effect of Tempo was *F*(1,4) = 14.12, *p* < .05, *η_p_^2^* = .78. The interaction effect of Pattern×Tempo was just above the significance threshold [*F*(1,4) = 7.29, *p* = .054, *η_p_^2^* = .65]. For *SDφ_finish_* the effect of Pattern was *F*(1,4) = 21.82, *p* = .01, *ηp2* = .85 and the effect of Tempo was *F*(1,4) = 13.34, *p* < .05, *ηp2* = .77. Crew coordination variability at the finish (*SDφ_finish_*) additionally showed a significant interaction effect of Pattern×Tempo [*F*(1,4) = 8.36, *p* < .05, *ηp2* = .68] indicating a significant decrease in coordinative variability from 20 to 30 *spm* for the antiphase pattern (*p* < .05). *post hoc* tests showed significant differences between antiphase coordination at 20 and 30 *spm* as the finish values show similar results. Note that the effect sizes are all above.65 (indicating large effects) but need to be interpreted with caution due to the low sample size (5 pairs) on which they are based.

The accuracy of crew coordination was calculated as the absolute error with respect to the intended pattern (e.g., the deviation from 0° for in-phase and 180° for antiphase in subplots 2A-D in Figure 1). Figure 2B suggests that antiphase coordination was performed less accurately than in-phase coordination (see Supplementary Table 3 for means and standard errors), though the repeated measures ANOVA on AE*ϕ* yielded no significant effects.

The drive-recovery ratio increased with stroke rate [*F*(1,4) = 54.30, *p* < .01, *η_p_2* = .93], indicating that drive and recovery became more equal in duration at 30 *spm* (reflected in a value closer to 1; see Supplementary Table 3 for means and standards errors). Further, an interaction effect of Pattern×Tempo [*F*(1,4) = 11.41, *p* < .05, *η_p_2* = .74] indicated that at 20 *spm* the drive-recovery ratio was significantly lower for in-phase than for antiphase, *post hoc* tests showed a significant difference between in- and antiphase coordination at 20 *spm* (*p* = .039, *d* = 1.35), but not at 30 *spm* (*p* = 0.314, *d* = −0.51).

### Surge velocity fluctuations

**Figure 3:**
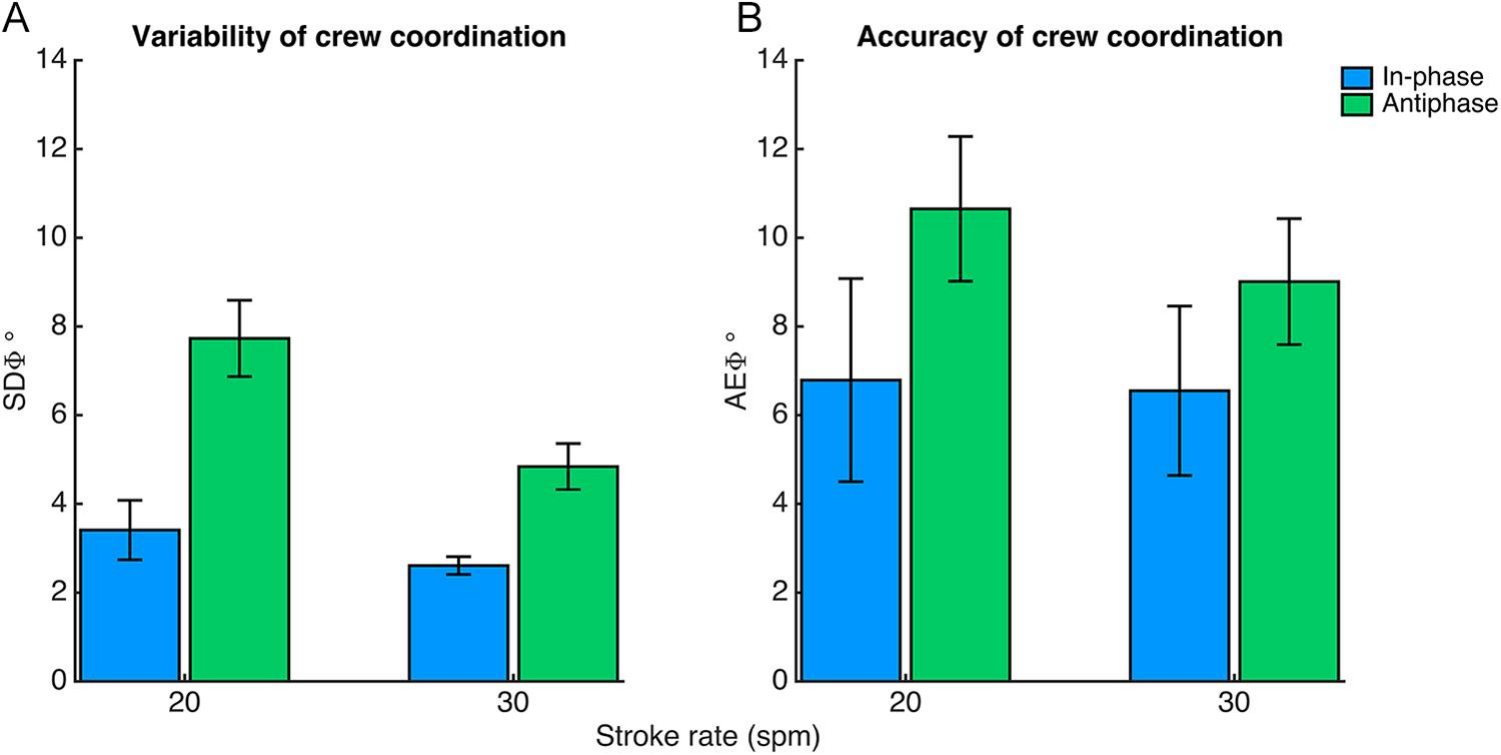
Average values of velocity fluctuations **(A)** and race times **(B)** for in- and antiphase at 20 and 30 spm, as determined over 5 out of 9 pairs (see ‘coordination breakdowns’). Error bars represent standard errors.

The examples of the obtained kinematic time series in Figure 1 show less surge velocity fluctuations for antiphase (subplots 1C and 1D) compared to in-phase rowing (subplots 1A and 1B), especially at 30 *spm* (subplots 1D vs. 1B). Indeed, the main effects of Pattern, *F*(1,4) = 25.98, *p* = .001, *η_p_^2^* = .96, and Tempo, *F*(1,4) = 103.97, *p* = .001, *η_p_^2^* = .96, obtained for *SDsurge* confirmed that within-cycle velocity fluctuations significantly reduced for rowing in antiphase compared to in-phase and increased with stroke rate (see Figure 3A). The reduction in surge velocity fluctuations when rowing in antiphase was even more pronounced at the higher stroke rate, as indicated by an interaction effect of Pattern × Tempo [*F*(1,4) = 112.87, *p* < .001, *η_p_^2^* = .97]. *post-hoc* tests showed significant differences for all pairwise comparisons (all *p*'s < .0125, −5.64 > *d* > −2.63). We verified that antiphase rowing indeed reduced surge velocity fluctuations by on average 19% at 20 *spm* to 30% at 30 *spm*. *SDheave* and *SDpitch* were higher at 30 than at 20 *spm*. The significant effect of Tempo was *F*(1,4) = 68.64, *p* = .001, *η_p_^2^* = .95 for *SDheave* and *F*(1,4) = 10.03, *p* < .05, *η_p_^2^* = .72 for *SDpitch*. *SDheave* and *SDpitch* were not significantly affected by Pattern. *SDroll* was not significantly affected by Pattern nor Tempo.

### Race time

All pairs were able to perform at the two instructed stroke rates and rowed at similar heart rates for in- and antiphase (as instructed), indicating that rowers produced a similar effort in both the in- and antiphase conditions. Figure 3B shows the 1,000 m race times as averaged over the five pairs. Evidently, race times were faster at 30 compared to 20 *spm* as supported by an effect of Tempo [*F*(1,4) = 77.46, *p* = .001, *η_p_^2^* = .95]. Figure 3B shows faster race times while rowing in in-phase with respect to antiphase coordination, which is supported by a main effect of Pattern [*F*(1,4) = 16.38, *p* < .05, *η_p_^2^* = .80].

## Discussion

The aim of this study was to test whether rowers were able to row in an antiphase pattern on the water. Next, we aimed to verify whether antiphase rowing on water indeed decreased surge velocity fluctuations and whether this would yield a higher boat speed and faster race times. We tested nine pairs rowing four 1,000 *m* trials in a four-person rowing boat that was modified for two rowers, while they rowed in in-phase and antiphase crew coordination at 20 and 30 *spm*. For an impression of the antiphase rowing on water, see Supplementary Video 1.

### Crew coordination

The rowers in the present study had never rowed in antiphase before. Still, already at these first attempts they managed quite well to row in this pattern. All nine pairs were able to row at least one antiphase trial without coordinative breakdowns. Five pairs were able to row in all trials without a single coordinative breakdown, while two other pairs only showed a coordinative breakdown once and were able to instantly restore coordination and continue rowing in antiphase for the rest of the trial (one pair while rowing at 20 *spm* and the other pair while rowing at 30 *spm*). For two of the nine pairs, antiphase coordination broke down repetitively within their 30 *spm* trial, while at 20 *spm* no breakdowns occurred. This observation does not fit well with the lower variability at 30 compared to 20 *spm* observed in pairs that did not show coordinative breakdowns. A lower variability is indicative of higher coordinative stability, suggesting more stable crew coordination at 30 *spm* in comparison to 20 *spm*. As such, these breakdowns seem unrelated to a frequency-related decrease of stability, and may have occurred due to a temporary perturbation or a loss of attention (5), as it has been shown that the degree of attention devoted to synchronising with a partner affects the stability of interpersonal coordination (9, 18). This remains to be investigated further, e.g., though experimental manipulation of attention (e.g., dual-task paradigm) or (e.g., mechanical) perturbations.

### Surge velocity fluctuations and race time

Antiphase rowing indeed reduced within-cycle velocity fluctuations by on average 19% at 20 *spm* to 30% at 30 *spm*, which theoretically reduces power losses due to drag in comparison to in-phase rowing (2, 4). Theoretically, the reduction in surge velocity fluctuations is optimised when the rowers move in exact antiphase relation throughout the whole stroke cycle; this would null their combined center of mass displacement with respect to the boat (5–7). However, it may be challenging to attune the drive- to the recovery phase of a crew member and vice versa if both phases differ in duration, especially at lower stroke rates (20 *spm*) where the recover lasts relatively longer than the drive phase (17). This is also supported by the findings that for both in- and antiphase crew coordination was less variable at 30 compared to 20 *spm* and that drive and recover became more equal in duration (as indicated by drive-recovery ratio closer to 1) at 30 *spm* in comparison to 20 *spm.* Indeed, the reduced surge velocity fluctuations for antiphase were especially pronounced at 30 *spm*, which further supports that on-water antiphase rowing becomes more beneficial when rowing at higher (racing) stroke rates (19). The difference in drive- and recover duration is less of an issue for in-phase crew coordination as here the rowers match their drive (and recovery) phases with one and other.

Rowing in antiphase resulted in a reduction in surge velocity fluctuations, but did not (yet?) result in faster racing times compared to in-phase coordination. Nevertheless, at high pace (30 *spm*) one of the pairs (of which the rowers both rowed at World Cup level) managed to row only 4 *s* slower in antiphase (3:44 *min*) compared to in-phase (3:40 *min*). This is a small difference, considering that this is the very first time for them to row in antiphase. Whether the latter generalises to other pairs rowing at World Cup level and whether more practice in antiphase may result in faster racing times, requires further research.

### Future directions

In the current experiment, most rowers were surprisingly quickly able to row in antiphase coordination in a stable way, even though it was their very first time. As the results obtained here provide a positive indication for rowers’ ability to learn to coordinate in an antiphase rowing pattern, it would be interesting to study to which degree rowers can improve crew coordination by practicing the antiphase pattern. Moreover, in antiphase the velocity of the boat remains quite constant throughout the cycle, as verified by our results. This suggests that rowing in antiphase is not just a matter of changing the timing or phasing of the drive and recovery phase with respect to the other rower(s), but also changes the coordination (e.g., in terms of force application) of the individual rowing movement itself. Hence, it likely implies that individual rowing technique may need to be optimised for antiphase rowing. Likewise, the design and rigging (i.e., the ‘settings’ of the boat such as oar length) may need to be optimised to account for the changes in boat velocity and distribution of power throughout the rowing cycle when rowing in antiphase. A next logical step therefore would be to investigate the effect of rowing in antiphase on the power production and force profiles of the rowers, through measuring forces applied on the oarlocks and footboards. More insight on the biomechanics of antiphase vs. in-phase crew rowing, such as on difference in hydrodynamics around the blades and the change in air resistance may attribute to find out if rowing in antiphase can truly be faster than rowing in in-phase (or not).

To investigate important next steps, such as the biomechanics of antiphase rowing, and antiphase coordination at higher, more realistic racing stroke rates, a specially built boat would be required. The use of a (relatively heavy) four-person instead of two-person boat (to allow for sufficient space for the oars not to collide in antiphase coordination) boat forms a limitation of the current experimental setup. Future research, using an experimental setup in which the dimensions of the boat more realistically match that of the rowing crew is needed to further test and verify the possibilities of antiphase rowing on racing stroke rates and its effects on boat movements and velocity. For instance for an eight, dividing the crew into two groups of four rowing in antiphase with each other would require (only) 70 *cm* extra space in the middle, to provide enough space for the oars not to collide with other crew members’ oars and backs (6). As boats can be built under minimum weight (and then precisely brought to minimum weight using lead strips), adding 70 *cm* to a 1990*cm* boat can probably be achieved at minimum weight. Such a boat would also make it possible to test antiphase rowing in competition. For now, antiphase rowing falls within the boundaries of the regulations of the International Rowing Federation (FISA), as it concerns a change of technique, not an innovation in equipment. Whether this will remain the case, or whether the technique will be banned, only time will tell.

## Conclusion

Despite that it was their first attempt, most pairs performed the unconventional antiphase pattern stably, even at high (racing) stroke rates. Antiphase rowing indeed reduced boat velocity fluctuations, especially at higher stroke rates, but did not yield faster racing times in these first attempts. Nonetheless, given the potency to improve antiphase rowing through practice and optimisation of the design and rigging of the boat, these results provide a promising first step in investigating the possibilities of antiphase rowing on water.

## Data Availability

The raw data supporting the conclusions of this article will be made available by the authors, without undue reservation.
