# Supplementary material for: Comparing in-phase to antiphase crew rowing: a first step from the lab to the water
Source: Front Sports Act Living. 2026 Apr 29;8:1604958. doi: 10.3389/fspor.2026.1604958 (PMC13169631; doi:10.3389/fspor.2026.1604958)
Supplement: Supplementary file 1 [file Datasheet1.pdf]

Supplementary Materials

Table 1. Participant characteristics.

Pair	Position	Gender	Age (years)	Category[a]	Height (m)	Weight (kg)	Rowing experience (years)
1	Stroke	M	21	HM	1.97	84.8	6
1	Bow	M	23	HM	1.98	85.9	5
2	Stroke	M	21	LM	1.86	78.3	9
2	Bow	M	24	LM	1.83	69.9	8
3	Stroke	F	20	LW	1.73	64.5	1
3	Bow	F	20	LW	1.70	57.6	2
4	Stroke	M	23	LM	1.78	70.7	2
4	Bow	M	21	LM	1.78	75.6	3
5	Stroke	F	20	HW	1.8	75.2	2
5	Bow	F	24	HW	1.72	84.4	6
6	Stroke	M	27	LM	1.85	70.7	18
6	Bow	M	26	LM	1.91	79.3	4
7	Stroke	F	37	HW	1.81	73.0	13
7	Bow	F	35	HW	1.74	60.8	10
8	Stroke	M	22	HM	1.95	90.9	3
8	Bow	M	21	HM	1.98	100.1	3
9	Stroke	F	18	HW	1.76	70.9	5
9	Bow	F	18	HW	1.80	70.9	6

[a] *H = open weight class, L = lightweight class, M = male, F = female.*

Table 2. Rigging and specifications of the boat and oars.

**Boat**	
Name:	Gyasterix (2002)
Type:	Hudson C4.31 Classic 4-/X
Weight[a]:	44.0 kg
Weight range rowers:	75-88 kg
**Oars**	
Type:	Croker S4
**Rigging**	
Span:	1.60 m
Oar Length:	2.88 m
Inboard:	0.88 m
Oar Angle:	+ 4 °

[a] *as used in experiment, i.e., including the measurement system and two riggers instead of four.*

Table 3. Means and standard errors of coordinative, boat, and heart rate measures for different conditions for 5 pairs.

Pattern	*In-phase*				*Antiphase*			
Stroke Rate	*20 spm*		*30 spm*		*20 spm*		*30 spm*	
Dependent Measure	*mean*	*SE*	*mean*	*SE*	*mean*	*SE*	*mean*	*SE*
SD DRP catch BB (°)	3.41	0.67	2.61	0.20	7.73	0.86	4.84	0.52
SD DRP finish BB (°)	4.81	0.87	3.01	0.40	10.89	1.15	6.55	0.92
SD DRP catch SB (°)	3.45	0.78	2.70	0.23	7.37	0.89	4.99	0.61
SD DRP finish SB (°)	4.28	0.60	3.49	0.27	11.64	0.75	6.94	0.76
AE DRP catch BB (°)	6.79	2.29	6.55	1.91	10.65	1.63	9.01	1.42
AE DRP finish BB (°)	6.48	0.69	7.27	1.95	11.03	1.48	13.30	2.32

AE DRP catch SB (°)	5.31	2.09	5.31	1.53	8.60	1.64	10.12	2.06
AE DRP finish SB (°)	9.69	1.63	9.79	2.86	11.77	1.14	12.61	1.87
ratio	0.69	0.03	0.98	0.02	0.74	0.02	0.95	0.04
stroke rate (spm)	19.97	0.26	28.44	0.26	20.66	0.13	28.40	0.09
SD surge (m/s^2)	1.58	0.11	2.46	0.15	1.28	0.08	1.77	0.14
SD heave (m/s^2)	0.43	0.03	0.60	0.02	0.42	0.02	0.61	0.02
SD pitch (°/s)	0.36	0.02	0.46	0.04	0.37	0.05	0.61	0.12
SD roll (°/s)	3.30	0.16	3.59	0.34	3.13	0.22	3.49	0.34
time (s)	263.00	8.09	235.00	8.14	276.80	7.43	247.60	9.55
mean HF stroke (bpm)[a]	161.28	7.46	178.61	1.83	152.38	4.97	175.40	2.60
mean HF bow (bpm)[b]	171.67	7.39	181.78	6.80	162.02	11.28	178.59	8.54

[a] *based on 4 pairs;* [b] *based on 3 pairs.*
